# Stress, Cognition, and Minority Health

**DOI:** 10.1093/geroni/igab046.288

**Published:** 2021-12-17

**Authors:** Toni Antonucci, Laura Zahodne, Melissa Gerald

## Abstract

This symposium examines the many factors influencing cognition and health among ethnically and racially diverse groups. Kindratt et al. use representative, national data to examine cognitive limitations and diabetes among foreign born Non-Hispanic Whites, Blacks, Hispanics, Asians and Arab Americans. Results indicate that prevalence of cognitive limitations was highest among non-Hispanic Whites and Arab-Americans, lowest among Blacks and Asians. Diminich et al. investigate the association of stressors and metabolic risk factors with cognitive/emotional functioning in a population of Hispanic/Latina(o) immigrants. They find a link between components of metabolic syndrome that are associated with domain specific deficits in cognition. These impairments are linked to posttraumatic stress, immigration related trauma and emotional health and wellbeing. Arevalo et al. examine cross-sectional and prospective associations of sleep duration and insomnia symptoms with measures of cognitive functioning among older Latinos from Puerto Rican ancestry with a longitudinal sample of older adults from the Boston Puerto Rican Health Study. Findings indicate that hours of sleep and insomnia symptoms are significantly associated with a number of global and specific cognitive factors. Finally, Munoz and colleagues, using a regional racially and ethnically diverse sample of people living in a large northeastern city, identified four stress profiles. These profiles (which focus on different types of stress) were differentially associated with working memory performance. In sum, these four papers document the experiences of stress and their association with cognitive functioning in diverse minority groups each of whom are disproportionately at risk for ADRD/RD. Gerald, from NIA, will serve as discussant.

